# Genetic variability of *Plasmodium falciparum* histidine-rich proteins 2 and 3 in Central America

**DOI:** 10.1186/s12936-019-2668-3

**Published:** 2019-01-31

**Authors:** Gustavo Fontecha, Alejandra Pinto, Denis Escobar, Gabriela Matamoros, Bryan Ortiz

**Affiliations:** Microbiology Research Institute, National Autonomous University of Honduras, Tegucigalpa, Honduras

**Keywords:** Malaria, pfhrp2, pfhrp3, *Plasmodium falciparum*, Rapid diagnostic test, Honduras, Guatemala, Nicaragua

## Abstract

**Background:**

Malaria is an important disease in many tropical countries. Rapid diagnostic tests (RDTs) are valuable tools for diagnosing malaria in remote areas. The majority of RDTs used for the diagnosis of *Plasmodium falciparum* are based on the detection of the specific histidine-rich proteins (PfHRP2 and PfHRP3). During the last decade, the threat posed by the lack of expression of these antigens and the variability of the proteins on the diagnosis of malaria has been widely discussed. The aim of this study was to evaluate the genetic diversity of *pfhrp2* and *pfhrp3* of *P. falciparum* isolates collected in three Central American countries.

**Methods:**

DNA samples were amplified and sequenced to assess the diversity of nucleotides and amino acids. A search for known epitopes within the amino acid sequence was carried out, and the sensitivity of the sequences was evaluated according to a predictive model. A phylogenetic analysis was carried out including homologous sequences from different regions of the world. Protein structures were predicted in silico.

**Results:**

Five different patterns for PfHRP2 and one pattern for PfHRP3 were identified. Isolates from Central America show a high level of genetic diversity in *pfhrp2;* however, the amino acid sequences seem to contain enough motifs to be detected by the RDTs currently available.

**Conclusion:**

It is unlikely that the variability of the *pfhrp2* and *pfhrp3* genes has a significant impact on the ability of the RDTs to detect the PfHRP antigens in Central America.

**Electronic supplementary material:**

The online version of this article (10.1186/s12936-019-2668-3) contains supplementary material, which is available to authorized users.

## Background

Malaria is a tropical disease responsible for a vast burden on public health in many developing countries. Although the incidence of malaria cases is still considerably high in Africa, Southeast Asia and the Amazon Basin, the Central American sub-region has experienced a notable decrease in the number of cases in recent decades [1]. These achievements have led to propose the elimination of malaria by the year 2030 by the countries of Central America and the island of Hispaniola [2].

Microscopy is the gold standard method for malaria diagnosis and has shown excellent performance when adequate infrastructure conditions and trained personnel are available [3]. However, due to the decrease in the number of cases in a geographic region, it is necessary to have diagnostic alternatives that do not require highly qualified personnel and equipment.

Rapid diagnostic tests (RDTs) were developed in the 1990s [4] and are valuable tools in the timely diagnosis of malarial infections in geographically remote areas. Currently available RDTs for the diagnosis of malaria are based on the detection of one or a combination of parasite antigens circulating in the blood of infected patients: the *Plasmodium falciparum* histidine-rich proteins PfHRP2 and PfHRP3, *Plasmodium* lactate dehydrogenase (pLDH), and *Plasmodium* aldolase [5]. PfHRP2 and PfHRP3 are the antigens most commonly used to detect *Plasmodium falciparum* infections and most of the RDT products (> 90%) available in the market use specific antibodies against both proteins [5–7]. PfHRP proteins are common target antigens for RDT manufacturing companies because of their abundance in blood during blood-stage infections [8], its specificity, and the presence of repetitive epitopes that enable their detection by multiple antibodies, increasing the sensitivity of the technique [9, 10]. PfHRP2 and its homologous PfHRP3 are soluble proteins encoded in genes with two exons interrupted by one intron, with a peptide signal encoded in exon 1 and a histidine-alanine rich repeat region in exon 2 [11, 12].

A recent study reported 21% of *P. falciparum* isolates from three Central American countries showing deletions of the exon 1-intron 1 segment of *pfhrp2* and *pfhrp3*, and 6.3% of the isolates lacked the exon 2 region of both genes [13].

Parasite failure to express the antigen is a well-demonstrated factor that affects the sensitivity of the PfHRP-based RDTs [14], but it has also been suggested that variations in the amino acid repeats within exon 2 of PfHRP2 could affect the sensitivity [15–17], which could have negative repercussions for malaria control and elimination programs. For this reason, a collection of parasite isolates from Central America was analysed to assess the genetic diversity within exon 2 of *pfhrp2* and *pfhrp3*, in order to provide useful information to better understand the possible effect of the diversity of parasites in the Central American region on the sensitivity of RDTs based on the detection of PfHRP antigens.

## Methods

### Sample collection

The aim of the study was to evaluate the genetic diversity within exon 2 of *pfhrp2* and *pfhrp3* genes of *P. falciparum* isolates collected in three Central American countries. A convenience sample was collected from a previous study that assessed the deletions of both genes and its flanking regions [13]. In this study, a subset of DNA samples that previously amplified the coding region of one or both genes were selected for further analysis. Thirty-five samples of *pfhrp2* (Honduras = 16, Nicaragua = 16, Guatemala = 3) and 5 samples of *pfhrp3* (Honduras = 3, Nicaragua = 2) were included. Samples showed heterogeneous parasitic densities. All the samples showed a previous positive result by a PfHRP2-based RDT. Since each of the three countries uses a different RDT brand for the routine diagnosis of malaria, the samples were analysed by the following RDTs: CareStart™ Malaria HRP2/pLDH(Pf/Pv) Combo (Honduras), SD Bioline MALARIA Ag P.f/P.v (Nicaragua) and CareStart™Malaria RDT Single Kit (Guatemala).

### DNA extraction

Blood samples of patients infected with malaria falciparum were collected on Whatman™ filter paper (GE Healthcare Bio-Sciences Corp, NJ, USA) for routine chloroquine resistance surveillance [18, 19]. DNA was extracted through a Chelex-100 based method [20]. A PCR method was used to confirm parasite species [3].

### DNA amplification, sequencing and in silico translation

**Fig. 1:**
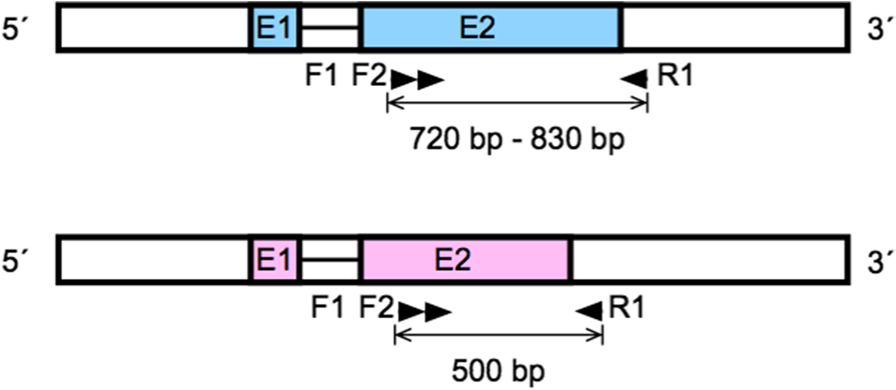
Scheme of the genes *pfhrp2* (blue) and *pfhrp3* (pink) showing the names and targets of the primers and sizes of the amplicons. Each gene is composed of two exons (E1 and E2) interrupted by a single intron

Exons 2 of the *pfhrp2* and *pfhrp3* genes were amplified by a semi-nested PCR approach [16] (Fig. 1). All amplification reactions were carried out in a volume of 50 µl, with 25 µl of 2X Master mix (Promega Corp.), 2.0 μl of each primer (Additional file 1) at a concentration of 10 μM and 1.0 μl of template DNA.

Reactions of both the primary and secondary PCR were carried out by an initial denaturation temperature at 94 °C for 10 min, followed by 35 cycles (37 cycles for the secondary PCR) of 94 °C for 50 s, an annealing step of 55 °C for 50 s, 72 °C for 1 min; and a final extension step at 72 °C for 10 min. The second amplification product was observed by agarose electrophoresis. Expected PCR products ranged between 720 and 830 bp for *pfhrp2* and < 500 bp for *pfhrp3*. Sequencing was performed with the forward and reverse primers of the secondary PCR at the Macrogen facilities (macrogenusa.com). Sequences were deposited at NCBI and accession numbers were assigned.

Geneious v.9.1.7 (Biomatters Ltd., Auckland, New Zealand) and Mega7 [21] v7.0.26 were used for sequence editing and analysis. The sequence length, nucleotide composition, and degree of polymorphism (π) were calculated. π was calculated as the average number of nucleotide differences per site between DNA sequences in all possible pairs in the sample population. Nucleotide sequences were translated in silico to corresponding amino acids (aa) using the correct open reading frame. Amino acid repeats were given a numeric code (1–20) according to previous reports [16, 22]. The number and frequency of each aa repeat were estimated. The number of type 2 repeats was multiplied by the number of type 7 repeats (2 × 7) in each sequence in order to predict the PfHRP2 detection sensitivity according to Baker’s regression model [15, 16]. Sequences were classified as “sensitive” if the score produced by the number of type 2 times type 7 repeats was greater than 43 and “non-sensitive” if it was less than 43 repeats. Minimal epitopes recognized by 11 PfHRP-specific monoclonal antibodies (MAbs) were searched and quantified in each PfHRP sequence [23].

The ClustalW tool was used to align the *pfhrp2*nucleotide sequences. The alignment was used to build a cladogram using the UPGMA (Unweighted Pair Group Method with Arithmetic Mean) method, with a bootstrap of 1000 replicates. A cladogram was constructed including only the sequences obtained in this study. A second set of cladograms was constructed including homologous sequences downloaded from the GenBank database at the National Center for Biotechnology Information (NCBI).

Sequences downloaded from NCBI were classified according to the geographical origin of the parasites based on four WHO Health Regions: Africa, Americas, Western Pacific, and South East Asia. Evolutionary distances were computed using the Maximum Composite Likelihood Method. Non-homologous sequences were selected as outgroups for the cladogram construction.

### Protein structure prediction

The secondary structure of PfHRP2 and PfHRP3 aa sequences was predicted through the template-based software RaptorX (http://raptorx.uchicago.edu). Sizes of partial aa sequences are shown in Table 1. The *p* value and the overall unnormalized global distance test (uGDT) were calculated for each model [24]. Two software were used to predict 3D models for the polypeptide sequences: (a) Protein Homology/analogy Recognition Engine v2.0 (PHYRE^2^) [25] (http://www.sbg.bio.ic.ac.uk/phyre2/html/page.cgi?id=index), and (b) I-TASSER (https://zhanglab.ccmb.med.umich.edu/I-TASSER/) [26].

**Table 1:** Length of nucleotide and amino acid sequences of *pfhrp2* and *pfhrp3*, number and geographic origin of isolates sharing each pattern, and accession numbers

Pattern	Size (bp)	Size (amino acids)	Number of isolates (%)	Honduras (%)	Nicaragua (%)	Guatemala (%)	Total (%)	Accession numbers NCBI
Pfhrp2 I	783	261	22 (62.9)	8 (22.9)	11 (31.4)	3 (8.6)	22 (62.9)	MH996688-90
Pfhrp2 II	720	240	4 (11.4)	–	4 (11.4)	–	4 (11.4)	MH996691
Pfhrp2 III	747	249	5 (14.3)	4 (11.4)	1 (2.9)	–	5 (14.3)	MH996692-3
Pfhrp2 IV	828	276	1 (2.9)	1 (2.9)	–	–	1 (2.9)	MH996694
Pfhrp2 V	828	276	3 (8.6)	3 (8.6)	–	–	3 (8.6)	MH996695
Total			35 (100.0)	16 (45.7)	16 (45.7)	3 (8.6)	35 (100.0)	
Pfhrp3 I	484	161	5 (100.0)	3 (60.0)	2 (40.0)	–	5 (100.0)	MH996696-7

## Results

### Nucleotide composition and diversity of *pfhrp2* and *pfhrp3* genes

Amplification products of 35 and 5 isolates were sequenced for *pfhrp2* and *pfhrp3* genes respectively. Five different *pfhrp2* patterns were identified (Accession Nos MH996688-95), while only one pattern was obtained for *pfhrp3* (MH996696-97) (Table 1). No correlation was observed between the nucleotide sequence and the geographical origin of the isolates. Pattern I of *pfhrp2* was the most frequently observed in the three countries (overall 62.9%). Only one parasite isolate had a unique *pfhrp2* sequence (pattern IV) and the remaining 4 sequence patterns were present in more than 3 isolates. As shown in Table 1, the size of the repeat sequence of exon 2 ranged from 720 to 828 nucleotides (240 to 276 aa) in *pfhrp2,* and it was shorter in *pfhrp3* with only 484 base pairs (161 aa). The most frequent aa within PfHRP2 sequences were Ala (42.4–44.4%), His (39.7–41%), and Asp (10.8–11.6%) (Additional file 2).

Although the five different patterns of *pfhrp2* revealed a similar nucleotide composition (Additional file 2), the nucleotide diversity (π) within the 35 isolates indicates this is a highly polymorphic gene (π = 0.044601). π was also calculated for 65 homologous *pfhrp2* sequences isolated from 4 WHO Health Regions with a result of 0.049603.

### Variation in amino acid repeat patterns

**Fig. 2:**
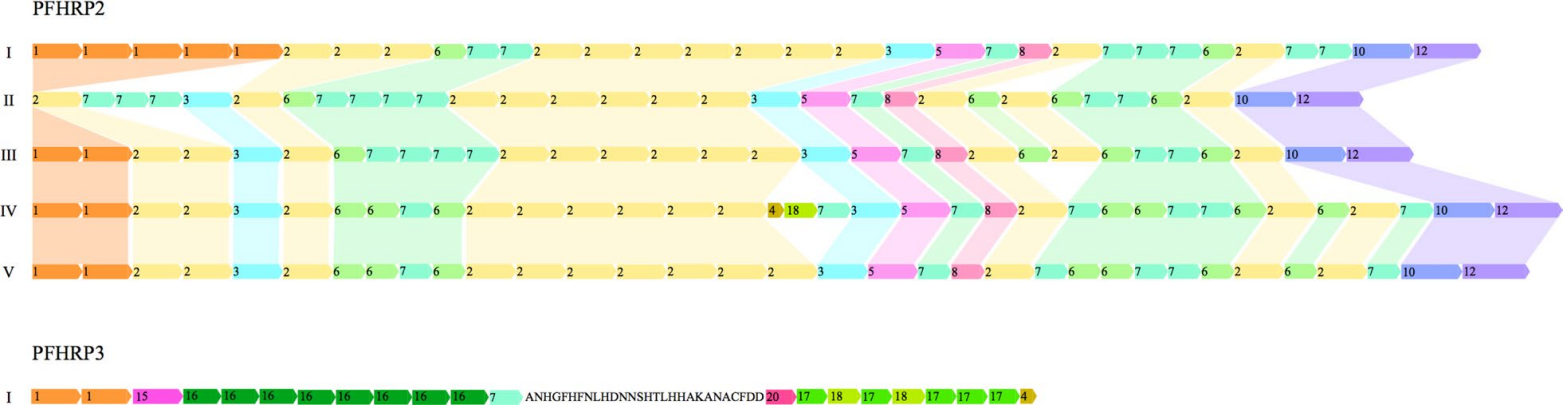
Comparison of repeat patterns in *pfhrp2* and *pfhrp3* obtained from parasites collected from Honduras, Nicaragua and Guatemala

Eleven and eight amino acid repeats were identified from *pfhrp2* and *pfhrp3* respectively (Table 2 and Fig. 2). Repeats 2, 3, 5—8, 10, and 12 were observed in all the patterns of *pfhrp2*. Repeat type 1 was absent from pattern II and repeats 4 and 18 were present only in pattern IV. Repeats 2 (36%) and 7 (25%) were the most frequent among the PfHRP2 sequences. All patterns, with the exception of pattern II, started with type 1 repeat and all patterns ended with the 10–12 repeat combination.

**Table 2:** Number and relative frequency of amino acid repeats of PfHRP2 and PfHRP3

Type	Repeat	Repeat frequency PfHRP2	Repeat (%) PfHRP2	Repeat frequency PfHRP3	Repeat (%) PfHRP3
1	AHHAHHVAD	128	11.67	10	10.0
2	AHHAHHAAD	395	36.0	–	–
3	AHHAHHAAY	48	4.37	–	–
4	AHH	1	0.09	5	5.0
5	AHHAHHASD	35	3.19	–	–
6	AHHATD	108	9.84	–	–
7	AHHAAD	276	25.15	5	5.0
8	AHHAAY	35	3.19	–	–
10	AHHAAAHHATD	35	3.19	–	–
12	AHHAAAHHEAATH	35	3.19	–	–
15	AHHAHHAAN	–	–	5	5.0
16	AHHAAN	–	–	40	40.0
17	AHHDG	–	–	25	25.0
18	AHHDD	1	0.09	5	5.0
20	SHHDD	–	–	5	5.0
Total		1097	100.0	100	100.0

The repeats’ pattern of the PfHRP3 gene showed a very different structure compared to PfHRP2. The most common repeat within PfHRP3 was type 16 (40%) followed by type 17 (25%). Types 2, 3, 5, 6, 8, 10, and 12 repeats were absent from PfHRP3, and type 7 represented only 5% of repeats. Motifs 15, 16, 17, and 20 were unique of PfHRP3 and absent from PfHRP2 sequences. A non-repetitive sequence of 28 amino acids interspersed between two blocks of repeats was present in all PfHRP3 sequences (ANHGFHFNLHDNNSHTLHHAKANACFDD) (Fig. 2).

### Prediction of sensitivity through the Bakers’ model

A predictive model proposed to assess the detection sensitivity of an RDT under parasite densities > 250 μl^−1^ was used for the 5 PfHRP2 patterns and the PfHRP3 sequence [16]. Baker’s model is based on a binary logistic regression as a function of the number of type 2× type 7 repeats and classifies a sequence as “sensitive” if the score or product of multiplication is higher than 43. The five PfHRP2 patterns produced scores ranging from 78 to 110. According to Baker’s predictive model, all the sequences could be detected with parasitaemia > 250 μl^−1^. The PfHRP3 sequence lacks type 2 repeats and reveals only one type 7 repetition.

### Epitopes targeted by monoclonal antibodies in RDTs

Linear epitopes (motifs) recognized by eleven MAbs [23] were searched in the sequences obtained in this study. Each MAb mainly recognize a dominant epitope (listed in Additional file 3) although they may be able to bind to other epitopes as well. The most frequent motif among the five patterns of PfHRP2 was AHHAADAHH, recognized by the MAb C1-13. This motif was present from 19 to 21 times in each pattern. The second and third most frequent motifs were AHHAHHA (14 to 16 times) and DAHHAADAHHA (7 to 10 times). Motifs HAHHAHHAADAHH and AYAHHAHHAAY were absent in all the patterns. Only two motifs (AHHAHHA and AHHAHHV) were present in the PfHRP3 pattern (Additional file 3).

### BLAST and cluster analyses

The five *pfhrp2* sequence patterns obtained in this study were compared with 65 similar sequences available in NCBI using the BLAST tool. The pattern I revealed 99% identity with the Honduran reference strain HB3 (Acc. No AY816261). Patterns II and III shared 97–99% similarity with Acc. No FJ871227, obtained from a strain isolated in Honduras. Patterns IV and V were 98% similar to the sequence KU723614 of Colombian origin. In contrast, the unique pattern of *pfhrp3* showed the highest similarity (99%) with a homologous sequence from Madagascar (Acc. No EU589824).

**Fig. 3:**
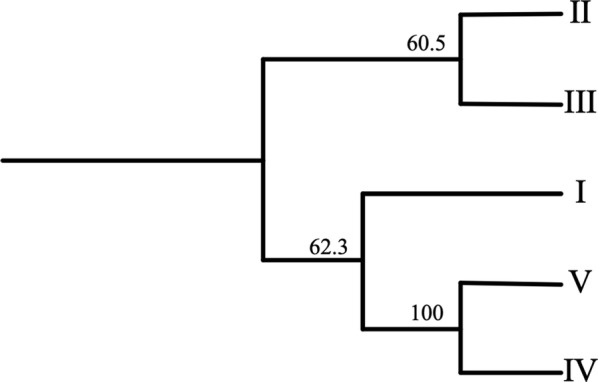
Cladogram clustering five *pfhrp2* sequence patterns (I–V) from *Plasmodium falciparum* isolates from Honduras, Nicaragua and Guatemala

The putative amino acid sequences corresponding to the five patterns of the *pfhrp2* gene from Central America were aligned in order to construct a cladogram that graphically revealed the similarities between them. As shown in Fig. 3, patterns IV and V revealed a high degree of similarity and their only difference was the insertion of a block of three repetitions (4-18-7) in the middle part of the pattern IV (Fig. 2).

**Fig. 4:**
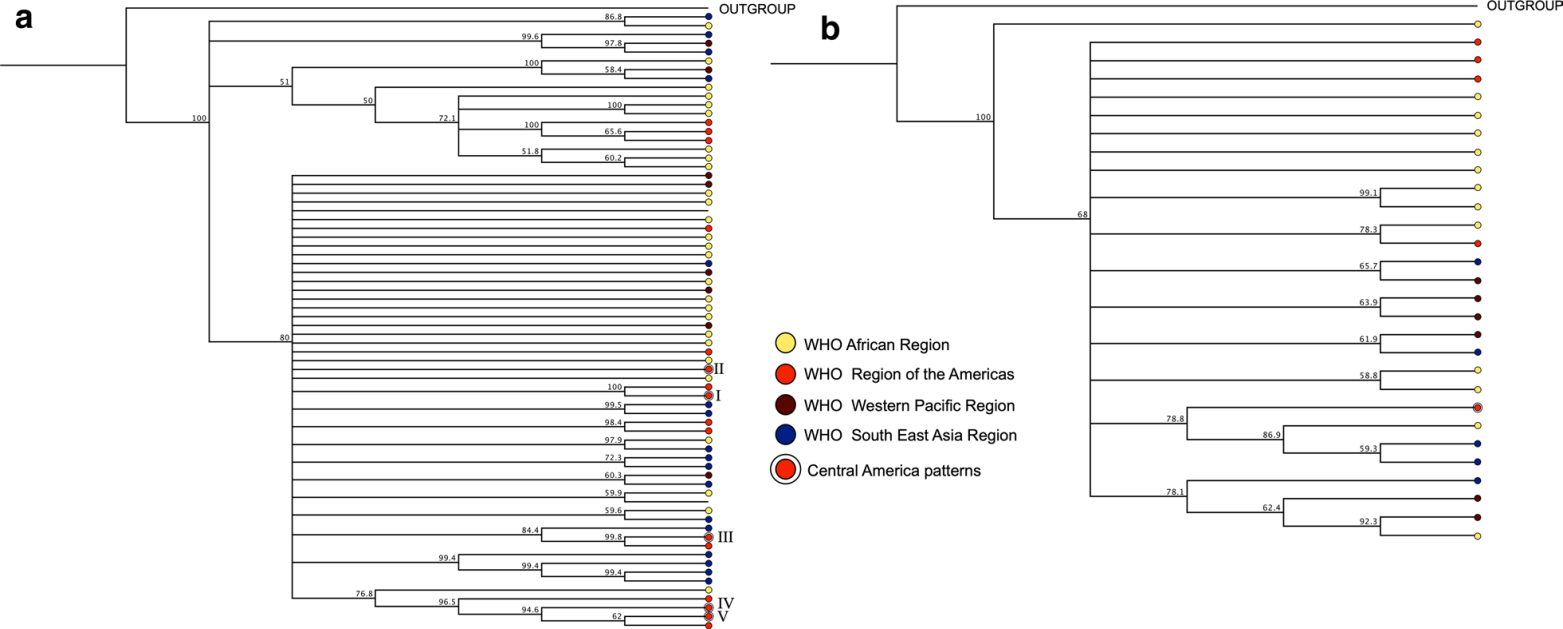
UPGMA cladograms showing the relationship between *hrp2* (**a**) and *hrp3* (**b**) sequences of *Plasmodium falciparum* isolates from Central America aligned with homologous sequences of parasites from other Regions. Colored dots indicate the geographical origin of the parasites by Health Region according to the WHO

For a better understanding of the similarities between *pfhrp2* and *pfhrp3* sequences obtained in this study and those from parasites from other regions of the world, 65 *pfhrp2* and 28 *pfhrp3* homologous sequences were downloaded from NCBI. Sequences were classified according to their geographical origin and according to WHO Health geographic Regions (for *pfhrp2*: African Region n = 27, Americas n = 11, Western Pacific n = 8, South East Asia n = 17, unknown n = 2; for *pfhrp3*: African Region n = 13, Americas n = 4, Western Pacific n = 7, South East Asia n = 4). A non-homologous sequence was included in each cladogram as an outgroup (Fig. 4). All sequences were aligned through ClustalW and a translation alignment method. Translation alignments were performed to nucleotide sequences that were translated into protein. Both alignments were used to construct separate cladograms for *pfhrp2* and *pfhrp3*.

No clear geographic clustering is observed with any of the two cladograms of both genes. However, sequences from Central America and sequences from some American countries cluster together for *pfhrp2*.

### Protein structure prediction

Based on their sequence and homology, the protein secondary structure was predicted for five partial PfHRP2 patterns and one PfHRP3 partial sequence, through an online template-based software. Partial amino acid sequences of exon 2 downloaded from NCBI were also analysed (Accession numbers AY816261 and KC558597). Complete coding sequences (CDS) including 305 amino acids for PfHRP2 (Acc. No XM_002808697) and 264 amino acids for PfHRP3 (Acc. No U69552) were also predicted for secondary structure using the RaptorX software.

As shown in the additional file 5, the predicted secondary structure for all the polypeptides matches with helices and coils. p-value and uGDT were calculated for each model. p-value is the likelihood of a predicted model being worse than the best of a set of randomly-generated models for a protein. Values < 10^−3^ are a good indicator of relative quality of a model. uGDT measures the absolute model quality. Values > 50 are a good indicator of quality. The p-values of all models were slightly higher than 10^−3^, and the uGDT values ranged between 28 and 75. Five models obtained an overall uGDT > 50, therefore the robustness of these predictions is partially reliable (Additional file 4).

Three dimensional models of protein sequences could not be meaningfully predicted with the Phyre2 software since 0% of residues modelled at > 90% confidence, and ~ 89% of each sequence was predicted disordered.

**Fig. 5:**
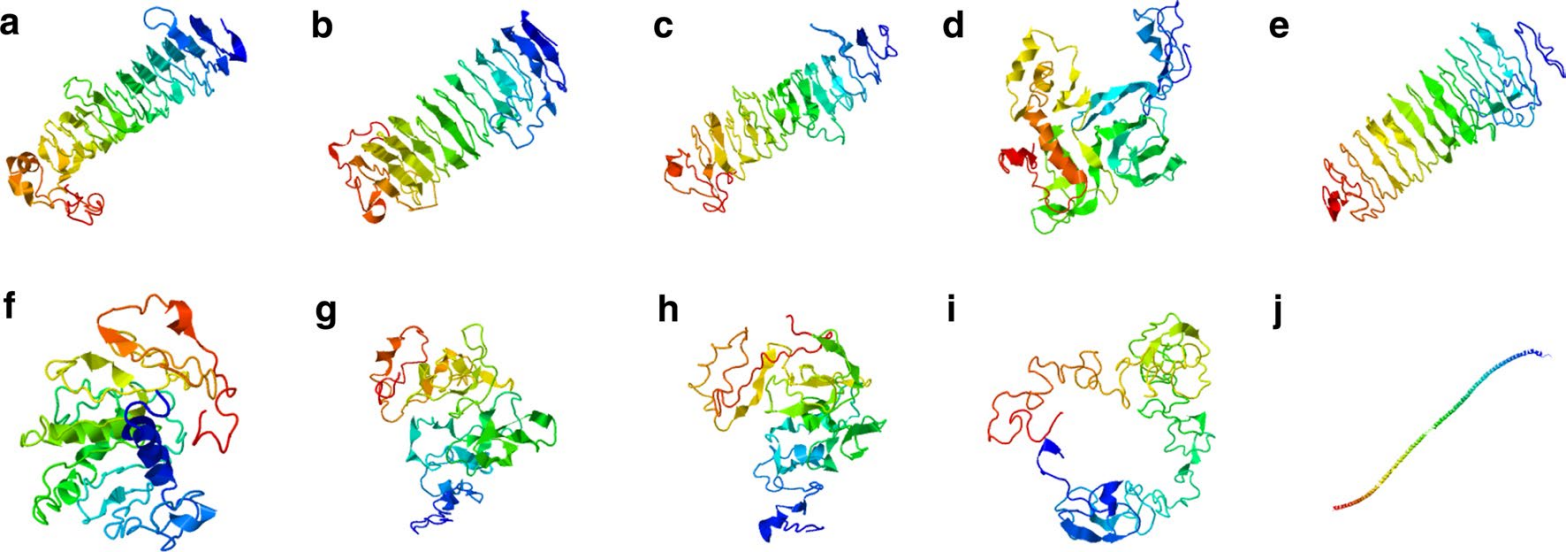
Template-based tertiary structure prediction of PfHRP2 (**a**–**e**) (Accession No XM_002808697), and PfHRP3 (**f**–**j**) (Accession No U69552) complete CDS proteins using the I-TASSER web server. Five models were included for each protein

The I-TASSER software was used to predict protein models for complete CDS of PfHRP2 (XM_002808697) and PfHRP3 (U69552). Five models were predicted for each polypeptide (Fig. 5). A C-score was calculated for each model. C-score is a confidence score for estimating the quality of predicted models. It is calculated based on the significance of threading template alignments and the convergence parameters of the structure assembly simulations. C-score is typically in the range of − 5 to 2, where a C-score of higher value signifies a model with a high confidence and vice versa.

The five PfHRP2 predicted models revealed C-scores ranged between − 2.78 to − 3.69 (Model 1: − 2.88, Model 2: − 2.87, Model 3: − 3.06, Model 4: − 3.13, and Model 5: − 3.69). For PfHRP3, the following C-scores were calculated for each model (Model 1: − 3.08, Model 2: − 3.32, Model 3: − 3.14, Model 4: − 2.86, and Model 5: − 2.41).

## Discussion

Four factors have been described as causes of failure for PfHRP-based RDTs for the diagnosis of *Plasmodium falciparum* infections: (a) Low parasitaemia, (b) gene deletions, (c) lack of antigen expression, and (d) polymorphisms in the amino acid sequences [17]. Previous studies reported the existence of parasite isolates with deletions in the *pfhrp2* and *pfhrp3* genes for the first time in Central America [13, 27]. This finding could hinder malaria control strategies relying on the detection of PfHRP2 and PfHRP3 antigens. In order to assess whether variations in the amino acid sequences of PfHRP2 and PfHRP3 could cause an added risk for the efficacy of the RDTs in Central America, the exon 2 of both genes was sequenced.

Five different *pfhrp2* sequences and a single *pfhrp3* sequence were identified from 35 and 5 *P. falciparum* isolates, respectively. According to this result, the isolates from Central America show a high level of genetic diversity in *pfhrp2* even though substantially lower than that reported in parasites from Africa and Asia. Sequence variations in both genes from isolates worldwide have been recently reviewed [28, 29]. When studying 458 isolates collected from 38 countries, African parasites showed greater diversity according to the proportion of unique PfHRP2 sequences per country. American isolates, on the other hand, were the least diverse. A similar trend was observed when *pfhrp3* sequences were studied from 80 isolates [29]. The highest levels of genetic variation (87%–98%) [15, 17, 29] were also reported in African countries in a review that included isolates from America, Africa and the Asia–Pacific region [28]. The nucleotide diversity (π) within sequences from Central America and worldwide confirmed that *pfhrp2* is highly polymorphic regardless of the geographic origin of the parasites [30].

The *pfhrp2* and *pfhrp3* genes codify for tandem repeats of amino acids. Tandem repeats are unstable genetic elements within genomes associated with rapid evolutionary changes [9]. *pfhrp2* and *pfhrp3* are located in the sub-telomeric/telomeric regions of chromosomes 8 and 13, respectively. Telomeric regions of *Plasmodium* are highly susceptible to changes due to homologous recombination [31, 32]. This could explain such a high gene diversity regardless of the geographic region [29]. In order to better understand the mechanisms of geographical dispersion of *P. falciparum*, an alignment of sequences obtained in this study was performed and compared with sequences of parasites from other regions of the world. A set of cladograms was constructed but no clear geographic clustering was observed. This apparent lack of clustering in the populations of the parasite suggests that *pfhrp2* and *pfhrp3* sequences are not under strong evolutionary selective pressure nor offer survival advantages for the species [29].

Some authors have hypothesized that variations in the sequence of PfHRP2 may affect the sensitivity of RDTs because of the polymorphisms of amino acid repeats [15, 29, 33]. Parasite isolates from Central America revealed eleven amino acid repeats in PfHRP2, all of them previously described in strains from other geographical regions [15, 22, 33–36]. The two most common motifs were type 2 (AHHAHHAAD) and type 7 (AHHAAD). The abundance of these motifs has been associated with an increase in the sensitivity of RDTs [15, 16, 36]. Both motifs have been proposed as potential epitopes targeted by monoclonal antibodies [16, 17, 23, 29] assuming that a higher epitope frequency within the sequence may result in greater sensitivity [23]. Bakers’ regression model [16] is based on the number of type 2 multiplied by the number of type 7 (2 × 7) repeats and it was applied in a case study from Uganda that reported a false negative RDT due to a low value of 2 × 7 repeats [37]. If this model has a good predictive capacity, the five PfHRP2 patterns described for Central American parasites could be detected even at low parasitaemia levels. However, there is evidence that suggests that Bakers’ model does not explain all the failures of non-detection of RDTs based exclusively on the score produced by the number of 2 × 7 repeats [17, 35, 38] and that the sequence diversity of repeats is not likely to negatively affect the performance of RDTs [29, 38]. Moreover, the amino acid sequence found in PfHRP3 is much less diverse than PfHRP2 which is consistent with reports elsewhere [15–17, 29, 36].

Specific antibodies against PfHRP2 cross react with PfHRP3 [12, 39, 40] but in circulating parasites from Central America this sequence lacks repeat 2 whilst repeat 7 is underrepresented. Furthermore, more than 90% of the isolates have deleted the loci associated to *pfhrp3* [13], therefore the expression of PfHRP3 does not seem to contribute significantly to the sensitivity of the RDTs in Central America.

A second approach was applied to attempt to correlate the amino acid sequences with the potential capacity of an RDT to detect the PfHRP2 antigen [41]. Eleven epitopes ranging from 8 to 15 amino acids which are recognized by commercially available monoclonal antibodies (MAbs) [23] were searched within the five PfHRP2 patterns and the unique PfHRP3 sequence. The five PfHRP2 sequence patterns revealed four motifs present at a high frequency, and five other motifs present in a lower proportion in almost all sequences (Additional file 3). The two most frequent epitopes were recognized by the MAbs C1-13 and 3A4. Similar results were reported in a study performed with 120 samples from Papua New Guinea [41]. Given the absence of two motifs (HAHHAHHAADAHH, AYAHHAHHAAY) within the sequences reported here, RDTs using MAbs C2-3 and Genway might be less sensitive, as suggested by Lee et al. [23]. In any case, it is important to remember that MAbs recognize dominant epitopes more avidly but in turn are able to recognize other epitopes with a certain degree of similarity to their dominant epitope. These results are further evidence that, despite the high genetic diversity, the RDTs based on the detection of PfHRP2 show an adequate sensitivity whenever the parasites express the antigen. The PfHRP3 sequence includes only 2 out of 11 motifs recognized by MAbs, consequently, this antigen could contribute less to the sensitivity of the RDT.

The three-dimensional structure of PfHRP2 and PfHRP3 have not been elucidated. Because sensitivity of an RDT depends on the binding of MAbs to specific epitopes, which can be linear or conformational, two softwares were applied to predict in silico the secondary and tertiary structure of the PfHRP proteins. The predicted structures could be useful to better understand the topographic availability of epitopes for MAbs.

According to the RaptorX software, uGDT values > 50 are good indicators of absolute model quality. The highest value was obtained for the complete coding sequence (CDS) of PfHRP2 (uGDT = 75). Four partial amino acid sequences encoded in exon 2 showed values > 50. These values suggest that these proteins could be organized mainly as helices as shown in Additional file 5. Additionally, folded protein models predicted by I-TASSER showed low C-scores limiting the robustness of the models.

Bioinformatic analysis are preliminary tools for predicting protein structures, however, the predicted models indicate that although some epitopes could be hidden inside the protein, there are enough epitopes exposed on the surface available for the binding of specific MAbs.

One limitation of the study is that all the samples were diagnosed as positive through a PfHRP2-based RDT, and different results could have been observed if samples with a negative RDT and a positive PCR or microscopy test would have been included. A second limitation of the study is the small number of samples analysed per country. According to the World Malaria Report 2018 [42], the percentage of samples analysed in relation to the number of cases of *P. falciparum* infections during the year in which the samples were collected was of 12.5% (16/128) for Honduras, 5.9% (3/51) for Guatemala, and 4.7% (16/342) for Nicaragua. This limitation could not be overcome due to logistical obstacles, and consequently the objective of the study did not have epidemiological connotations.

## Conclusion

Despite these limitations this study provides insights into the genetic variability of *pfhrp2* and *pfhrp3* in isolates from three countries in Central America and shows that PfHRP2 is highly polymorphic. It is unlikely that this variability has a significant impact on the sensitivity of currently available PfHRP-based RDTs.

## Additional files

**Additional file 1.** List of the primer’s sequences used to amplify the exon 2 of *pfhrp2* and *pfhrp3.*

**Additional file 2.** Nucleotide and amino acid composition of five *pfhrp2* and 1 *pfhrp3* sequences.

**Additional file 3.** Frequency of epitope motifs present in the sequences of PfHRP2 and PfHRP3.

**Additional file 4.** Template-based secondary structure prediction of PfHRP2 (a–f), and PfHRP3 (g–h) partial proteins using the RaptorX web server. Letters (a–e) show the five patterns of PfHRP2 described in this study; (f) partial sequence of *Plasmodium falciparum* reference strain 3D7 (Accession No XM_002808697); (g) PfHRP3 sequence from Central American isolates; (h) *Plasmodium falciparum* strain N569 (Accession No KC558597); (i) Complete CDS of PfHRP2 from *Plasmodium falciparum* strain 3D7 (Accession No XM_002808697); (j) Complete CDS of PfHRP3 from *Plasmodium falciparum* accession No U69552. The boxes on the right indicate the prediction of the secondary structure. The red boxes show the probability of helix structures and the grey boxes indicate the probability of coil formation for each amino acid position.

**Additional file 5.** p-value and uGDT of secondary structures of models predicted for PfHRP2 and PfHRP3.

## Abbreviations

RDT: rapid diagnostic test
pfhrp2: *Plasmodium falciparum* histidine-rich protein 2 gene
pfhrp3: *Plasmodium falciparum* histidine-rich protein 3 gene
PfHRP: *Plasmodium falciparum* histidine rich protein
pLDH: *Plasmodium* lactate dehydrogenase
PCR: polymerase chain reaction
MAbs: monoclonal antibodies
UPGMA: unweighted pair group method with arithmetic mean
NCBI: National Center for Biotechnology Information
WHO: World Health Organization
uGDT: unnormalized global distance test
Acc. No: accession number
BLAST: Basic Local Alignment Search Tool
CDS: complete coding sequences
