# Supplementary material for: Genetic variability of Plasmodium falciparum histidine-rich proteins 2 and 3 in Central America
Source: Malar J. 2019 Jan 31;18:31. doi: 10.1186/s12936-019-2668-3 (PMC6357481; doi:10.1186/s12936-019-2668-3)
Supplement: Supplementary file 1 — Additional file 1. List of the primer’s sequences used to amplify the exon 2 of pfhrp2 and pfhrp3. [file 12936_2019_2668_MOESM1_ESM.docx]

| Primer name | Sequence |
| --- | --- |
| Pfhrp2-F1 | 5´-CAAAAGGACTTAATTTAAATAAGAG-3´ |
| Pfhrp2-R1 | 5´-AATAAATTTAATGGCGTAGGCA-3´ |
| Pfhrp2-F2 | 5’- ATTATTACACGAAACTCAAGCAC-3’ |
| Pfhrp3-F1 | 5´- AATGCAAAAGGACTTAATTC-3´ |
| Pfhrp3-R1 | 5´- TGGTGTAAGTGATGCGTAGT-3´ |
| Pfhrp3-F2 | 5’- AAATAAGAGATTATTACACGAAAG -3’ |
