# Supplementary material for: Genetic variability of Plasmodium falciparum histidine-rich proteins 2 and 3 in Central America
Source: Malar J. 2019 Jan 31;18:31. doi: 10.1186/s12936-019-2668-3 (PMC6357481; doi:10.1186/s12936-019-2668-3)
Supplement: Supplementary file 2 — Additional file 2. Nucleotide and amino acid composition of five pfhrp2 and 1 pfhrp3 sequences. [file 12936_2019_2668_MOESM2_ESM.docx]

| Sequence pattern | T(U) | C | A | G | His (%) | Ala (%) | Asp (%) |
| --- | --- | --- | --- | --- | --- | --- | --- |
| pfhrp2 I | 21.7 | 36.5 | 23.0 | 18.8 | 107 (41) | 112 (42.9) | 29 (11.1) |
| pfhrp2 II | 20.6 | 37.4 | 23.3 | 18.8 | 95 (39.7) | 107 (44.4) | 27 (11.3) |
| pfhrp2 III | 21.0 | 37.2 | 23.3 | 18.5 | 101 (40.6) | 108 (43.4) | 27 (10.8) |
| pfhrp2 IV | 20.7 | 37.3 | 23.7 | 18.4 | 111 (40.2) | 117 (42.4) | 32 (11.6) |
| pfhrp2 V | 20.7 | 37.4 | 23.6 | 18.4 | 111 (40.2) | 118 (42.8) | 31 (11.2) |
| Average | 20.9 | 37.2 | 23.4 | 18.5 |  |  |  |
| pfhrp3 I | \| 22.7 \|  \|  \|  \| \| --- \| --- \| --- \| --- \| | 30.6 | 28.5 | 18.2 | 55 (34.2) | 52 (32.3) | 18 (11.2) |
