# Supplementary material for: Genetic variability of Plasmodium falciparum histidine-rich proteins 2 and 3 in Central America
Source: Malar J. 2019 Jan 31;18:31. doi: 10.1186/s12936-019-2668-3 (PMC6357481; doi:10.1186/s12936-019-2668-3)
Supplement: Supplementary file 3 — Additional file 3. Frequency of epitope motifs present in the sequences of PfHRP2 and PfHRP3. [file 12936_2019_2668_MOESM3_ESM.docx]

|  |  | PfHRP2 pattern | | | | | PfHRP3 pattern | |  |
| --- | --- | --- | --- | --- | --- | --- | --- | --- | --- |
| Monoclonal antibody[23] | Epitope motif | I | II | III | IV | V | | I | |
| 3A4 | AHHAHHA | **14** | **14** | **15** | **16** | **16** | | 1 | |
| 2G12-1C12 | DAHHAADAHH | **8** | **10** | **7** | **7** | **7** | | 0 | |
| 1E1-A9 | AHHAHHV | 5 | 0 | 2 | 2 | 2 | | 2 | |
| A6-4 | HATDAHH | 3 | 4 | 4 | 7 | 8 | | 0 | |
| C1-13 | AHHAADAHH | **20** | **21** | **19** | **19** | **20** | | 0 | |
| N7 | DAHHAADAHHA | **8** | **10** | **7** | **7** | **7** | | 0 | |
| PTL-3 | YAHHAHHA | 2 | 3 | 3 | 3 | 3 | | 0 | |
| S2-5 | AHHASDAHHA | 1 | 1 | 1 | 1 | 1 | | 0 | |
| TC-10 | TDAHHAADAHHAADA | 1 | 2 | 2 | 1 | 1 | | 0 | |
| C2-3 | HAHHAHHAADAHH | 0 | 0 | 0 | 0 | 0 | | 0 | |
| Genway | AYAHHAHHAAY | 0 | 0 | 0 | 0 | 0 | | 0 | |
