# Supplementary figures and images for: Genetic variability of Plasmodium falciparum histidine-rich proteins 2 and 3 in Central America
Source: Malar J. 2019 Jan 31;18:31. doi: 10.1186/s12936-019-2668-3 (PMC6357481; doi:10.1186/s12936-019-2668-3)

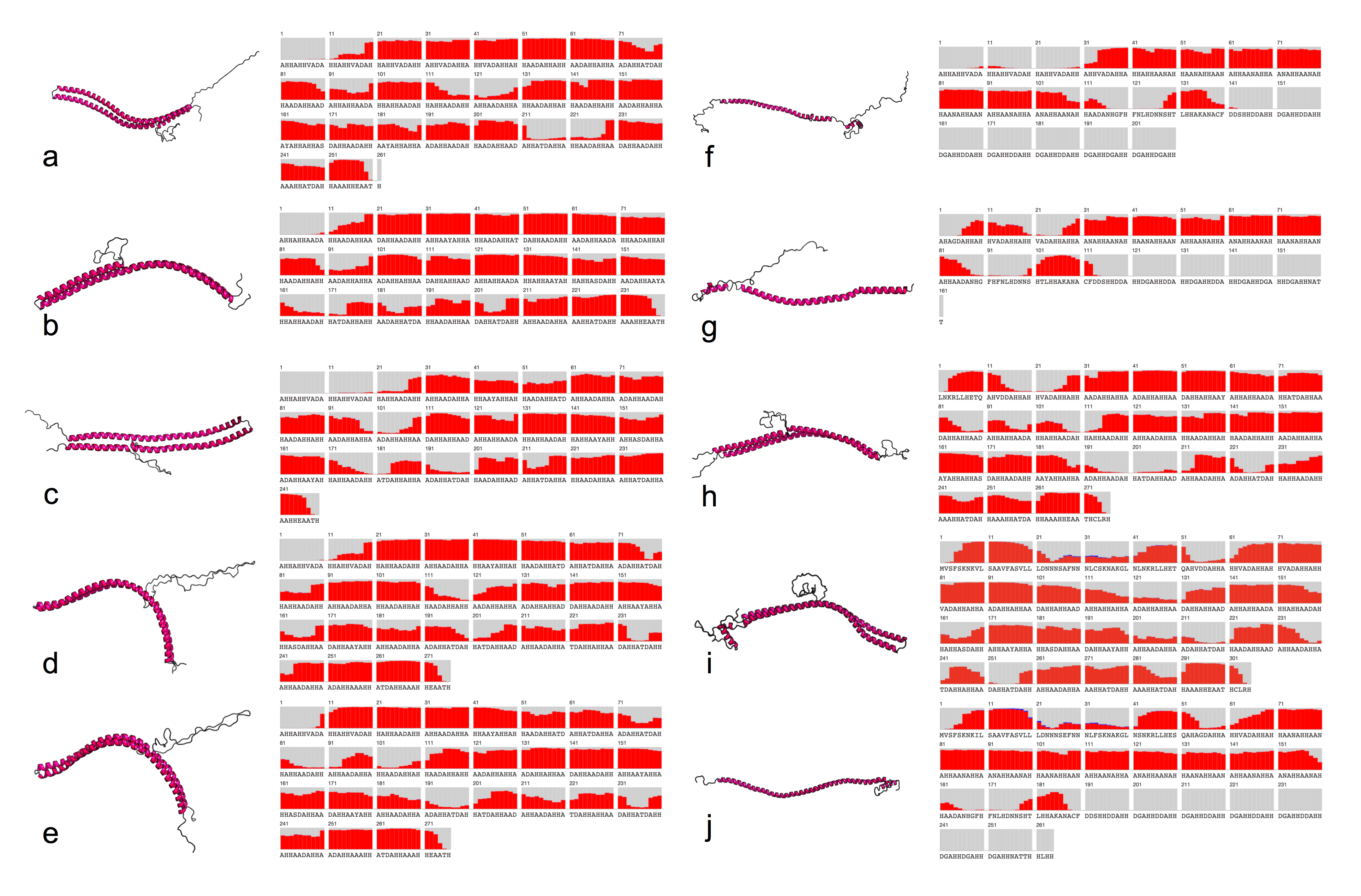

Supplement: Supplementary file 4 — Additional file 4. Template-based secondary structure prediction of PfHRP2 (a–f), and PfHRP3 (g–h) partial proteins using the RaptorX web server. Letters (a–e) show the five patterns of PfHRP2 described in this study; (f) partial sequence of Plasmodium falciparum reference strain 3D7 (Accession No XM_002808697); (g) PfHRP3 sequence from Central American isolates; (h) Plasmodium falciparum strain N569 (Accession No KC558597); (i) Complete CDS of PfHRP2 from Plasmodium falciparum strain 3D7 (Accession No XM_002808697); (j) Complete CDS of PfHRP3 from Plasmodium falciparum accession No U69552. The boxes on the right indicate the prediction of the secondary structure. The red boxes show the probability of helix structures and the grey boxes indicate the probability of coil formation for each amino acid position. [file 12936_2019_2668_MOESM4_ESM.png]
