# Supplementary material for: Genetic variability of Plasmodium falciparum histidine-rich proteins 2 and 3 in Central America
Source: Malar J. 2019 Jan 31;18:31. doi: 10.1186/s12936-019-2668-3 (PMC6357481; doi:10.1186/s12936-019-2668-3)
Supplement: Supplementary file 5 — Additional file 5. p-value and uGDT of secondary structures of models predicted for PfHRP2 and PfHRP3. [file 12936_2019_2668_MOESM5_ESM.docx]

| Sequence | p-value | uGDT |
| --- | --- | --- |
| PfHRP2 pattern I | 3.73 X 10^-3^ | **51** |
| PfHRP2 pattern II | 7 X 10^-3^ | **55** |
| PfHRP2 pattern III | 3.6 X 10^-3^ | 48 |
| PfHRP2 pattern IV | 7.06 X 10^-3^ | **55** |
| PfHRP2 pattern V | 7.87 X 10^-3^ | **54** |
| PfHRP2 3D7 partial gene AY816261 | 5.27 X 10^-3^ | 49 |
| PfHRP2 3D7 CDS XM_002808697 | 8.97 X 10^-3^ | **75** |
| PfHRP3 pattern I | 4.04 X 10^-3^ | 28 |
| PfHRP3 partial gene KC558597 | 4.75 X 10^-3^ | 48 |
| PfHRP3 CDS U69552 | 2.8 X 10^-3^ | 41 |
